# On the Dependence of Rheology of Hyaluronic Acid Solutions and Frictional Behavior of Articular Cartilage

**DOI:** 10.3390/ma13112659

**Published:** 2020-06-11

**Authors:** David Rebenda, Martin Vrbka, Pavel Čípek, Evgeniy Toropitsyn, David Nečas, Martin Pravda, Martin Hartl

**Affiliations:** 1 Faculty of Mechanical Engineering, Brno University of Technology, 616 69 Brno, Czech Republic; Martin.Vrbka@vut.cz (M.V.); Pavel.Cipek@vut.cz (P.Č.); David.Necas@vut.cz (D.N.); Martin.Hartl@vut.cz (M.H.); 2 Contipro a.s., Dolní Dobrouč 401, 561 02 Dolní Dobrouč, Czech Republic; Evgeniy.Toropitsyn@contipro.com (E.T.); Martin.Pravda@contipro.com (M.P.)

**Keywords:** articular cartilage, hyaluronic acid, rheology, friction

## Abstract

Hyaluronic acid (HA) injections represent one of the most common methods for the treatment of osteoarthritis. However, the clinical results of this method are unambiguous mainly because the mechanism of action has not been clearly clarified yet. Viscosupplementation consists, inter alia, of the improvement of synovial fluid rheological properties by injected solution. The present paper deals with the effect of HA molecular weight on the rheological properties of its solutions and also on friction in the articular cartilage model. Viscosity and viscoelastic properties of HA solutions were analyzed with a rotational rheometer in a cone–plate and plate–plate configuration. In total, four HA solutions with molecular weights between 77 kDa and 2010 kDa were tested. The frictional measurements were realized on a commercial tribometer Bruker UMT TriboLab, while the coefficient of friction (CoF) dependency on time was measured. The contact couple consisted of the articular cartilage pin and the plate made from optical glass. The contact was fully flooded with tested HA solutions. Results showed a strong dependency between HA molecular weight and its rheological properties. However, no clear dependence between HA molecular weight and CoF was revealed from the frictional measurements. This study presents new insight into the dependence between rheological and frictional behavior of the articular cartilage, while such an extensive investigation has not been presented before.

## 1. Introduction

An articular cartilage is a kind of hyaline cartilage which covers sliding surfaces in large synovial joints (e.g., the hip or knee). Under physiological conditions, the articular cartilage creates sliding surfaces with extremely low friction and minimal wear. It can also absorb impact loads quite well. The cartilage structure consists of a fluid and solid phase, which determine its mechanical properties [1]. A solid phase is composed of an extracellular matrix from collagen fibrils and proteoglycans. According to the orientation of collagen fibrils, the cartilage structure can be divided into several layers: superficial, middle, deep and calcified zones [2]. Collagen fibrils are oriented tangentially to the cartilage surface in the superficial zone whereas they are mostly perpendicular to the surface in the deep zone. The collagen content is the highest in the superficial zone and decreases towards the deep zone. On the contrary, the proteoglycan content is the lowest in the superficial zone. The superficial zone also has the highest porosity, which means the highest content of fluid phase. This interstitial fluid is mainly composed of water and electrolytes [3].

A cartilage-on-cartilage motion exhibits very low friction under physiological conditions. However, unhealthy lifestyle, obesity, or traumatic injuries can lead to the damage of the articular cartilage and can cause diseases such as osteoarthritis, chondropathy, etc. Osteoarthritis is one of the most common diseases of the musculoskeletal system. These days, it afflicts about 70% of people older than 70 years [4]. Osteoarthritis is characterized by an imbalance between the synthesis and wear of the articular cartilage. The surface of the cartilage shows areas of softening, fibrillations, or erosions. In the later stage, there may even be areas of cartilage loss. This cartilage damage leads to a distraction of the cartilage lubrication mechanism and a higher friction. Progression of osteoarthritis is also connected with changes in the composition of the synovial fluid [5]. Osteoarthritic synovial fluid is diluted by inflammatory effusion and the concentration of the individual components is changing.

Viscosupplementation is one of the noninvasive methods for the curing of osteoarthritis. This method consists of intra-articular injections with hyaluronic acid (HA) into the joint capsule. The original theory about viscosupplementation [6] assumed the improvement of the rheological properties of synovial fluid by exogenous HA. Higher viscosity and improved viscoelastic properties should lead to better lubrication and lower friction of the osteoarthritic articular cartilage. Exogenous HA can be detected in the synovial fluid for only a few days after the injection but medical studies [7] reported positive effects of this treatment method even after 6 months. This pointed out another physiological effect of this treatment method such as a synthesis of endogenous HA or an anti-inflammatory effect [8]. However, the mechanism of action of viscosupplementation has not been sufficiently clarified yet.

Hyaluronic acid (HA) is a polymer of disaccharides composed of D-glucuronic acid and N-acetyl D-glucosamine. It is one of the primary constituents of synovial fluid. In a healthy synovial joint, the concentration varies between 1 and 4 mg/mL [9,10], and the molecular weight ranges from 4 kDa up to 8 MDa [11]. HA is the main constituent which affects the rheology of synovial fluid [12]. Concentration and molecular weight are the key parameters which affect the viscosity and viscoelastic properties of HA solutions. Solutions with a higher concentration exhibit a higher viscosity [13,14], the same as solutions with higher molecular weights [13,15]. Solutions with higher molecular weights also report higher values of the storage (G’) and loss (G’’) modulus [13,15], and the value of crossover frequency is decreasing [14]. Longer polymer chains need more time to disentangle so the molecular transition from a predominantly viscous response to an elastic response occurs at lower frequencies. In osteoarthritic synovial fluid, the concentration and molecular weight of HA is decreased [16]. Exogenous HA which is injected into the joint capsule during viscosupplementation, should restore the rheological properties of healthy synovial fluid. Due to the low concentration and molecular weight of endogenous HA, the rheological properties of mixed synovial fluid with viscosupplement are primarily dependent on exogenous HA. The best results are obtained for synovial fluids mixed with cross-linked HA [17,18].

The superior tribological properties (low friction and minimal wear) of the articular cartilage under physiological conditions seem to be established by an interaction between the solid and fluid phase. However, a detailed cooperation between the cartilage structure and the synovial fluid has not been clarified yet. The superior tribological performance was attributed to many lubrication modes such as boundary lubrication [19], weeping lubrication [20], or micro-elastohydrodynamic lubrication [21]. Human synovial joints operate under variable loading and motions including rolling and sliding during various daily activities. Therefore, the lubrication of natural synovial joints is likely to be actualized not by a single lubrication mode but by a synergistic combination of them. In recent years, theories about adaptive multimode lubrication [22,23], biphasic lubrication [24], or hydration lubrication [25] have been published.

The importance of HA within these various lubrication regimes of the articular cartilage was already proved. HA solutions showed lower values of coefficient of friction compared to the simple solutions such as phosphate buffer saline (PBS) [26] or Ringer’s solution [4]. Unlike the rheology, the interaction between HA and other synovial fluid constituents plays an important role. A mixture of HA and phospholipids [27] leads to a lower friction compared to a simple phospholipid solution. Surface-anchored HA molecules complex synergistically with lipids present in the synovial fluid to form a boundary lubricating layer with very low friction (*µ* ≈ 0.001) [28]. Molecules of protein γ-globulin and HA have a different electric charge so their molecules attract each other and form complex structures, which contributes to the lower friction [29,30]. On the other hand, albumin and HA have the same electric charge and they repel each other. This interaction is not useful for the reduction of friction [29,30].

The HA molecular weight has a significant impact on the rheology of synovial fluid. Rheological studies have shown that HA with a higher molecular weight exhibits higher viscosity and better viscoelastic properties. HA also plays an important role in the reduction in friction in the osteoarthritic joint. Tribological studies showed a significant decrease in friction after the addition of HA to the tested lubricant and also the importance of HA in the formation of boundary lubricating layers on the cartilage surface. However, so far, no one has focused on how the HA molecular weight affects the friction of the articular cartilage. Therefore, this study is focused on the changes in cartilage friction caused by differences in HA molecular weight. The investigation is based on the combination of the detailed rheology description of HA-based solutions along with its impact on the frictional behavior of the articular cartilage. According to the author’s best knowledge, such methodology, possibly bringing an important implication for clinical practice, has not been applied before. Viscosupplementation is a procedure which is commonly used to cure osteoarthritis in many human joints but results reported by patients show substantial differences. Therefore, it is important to examine how the main constituent of viscosupplement (i.e., HA) affects the friction of the articular cartilage.

## 2. Materials and Methods 

**Figure 1:**
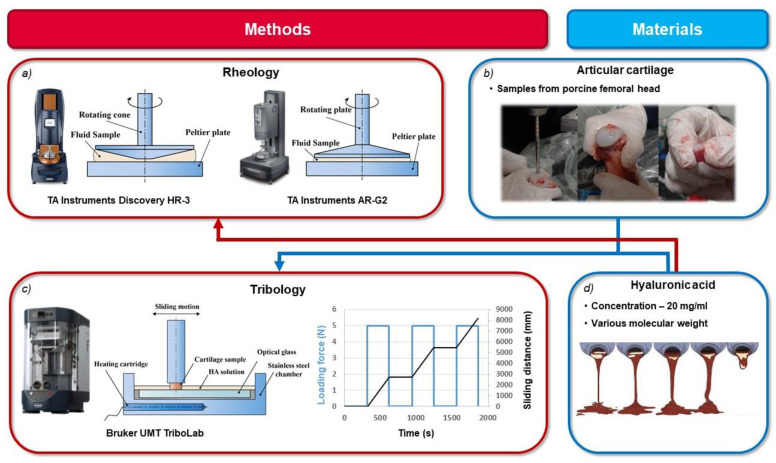
Scheme of research plan: (**a**) rheological measurements; (**b**) cartilage sample preparation; (**c**) frictional measurements; (**d**) tested solutions.

The rheological properties of tested hyaluronan solutions were determined using a TA Instruments Discovery HR-3 rheometer (TA Instruments, New Castle, DE, USA, Figure 1a). Experiments were conducted using a stainless-steel cone and plate geometry (60 mm diameter cone with a 1° cone angle). The temperature was set to 37 °C during all experiments. In the steady shear test, the shear rates ranging from 0.01 to 5000 s^‒1^ were applied to the tested fluids. Dependency of viscosity on shear rate (viscosity curves) was evaluated. The viscoelastic properties of the tested solutions with a higher viscosity were analyzed by performing a small-amplitude oscillatory shear test (SAOS) using a TA Instruments AR-G2 rheometer (TA Instruments, New Castle, DE, USA) Figure 1a) in a plate–plate configuration (20 mm diameter plate). The SAOS test measures the elastic and viscous modulus, when the tested material is subjected to sinusoidal strain. Frequency sweep measurements were conducted at 5% strain over a frequency range of 0.05–5 Hz. Frequency sweeps were performed at strain amplitude which was determined to be in the linear viscoelastic range. Temperature was set to 37 °C. Dynamic moduli were determined as a function of the angular frequency. All experiments were repeated three times with a fresh sample of HA. From these data, average values and standard deviations were counted. 

In an effort to understand the effect of HA molecular weight on the rheology and friction of cartilage, all experiments were performed using simple HA solutions with different molecular weights. In total, four HA solutions with a concentration of 20 mg/mL and a molecular weight of 77 kDa, 640 kDa, 1060 kDa and 2010 kDa were tested. Solutions were prepared from HA powder (Contipro, Dolní Dobrouč, Czech Republic) by dissolution of the required amount of powder in PBS. The solution was stirred by a magnetic stirrer and heated to 60 °C for at least three hours to ensure the proper dissolution of HA. 

The reciprocating sliding tests were conducted on a commercial tribometer Bruker UMT TriboLab (Bruker, Billerica, MA, USA) in a pin-on-plate configuration (Figure 1c). The coefficient of friction was investigated as a function of time for the sliding pair of the stationary glass plate made from the optical glass B270 and the moving porcine cartilage specimen. This specimen was loaded with a constant load of 5 N. The sliding speed of 10 mm/s was selected and the reciprocating stroke was 20 mm. The contact was fully flooded with HA solution. To mimic the temperature of the human body, the lubricant was heated to 37 °C via heating cartridges in a steel chamber. Before each experiment, an unloaded cartilage sample was immersed in lubricant for 320 s to let the cartilage soak with lubricant. At the end of this preliminary phase, the cartilage was loaded and the friction test started immediately. After 300 s (75 cycles, sliding distance of 2740 mm), the sliding test was interrupted and the cartilage was unloaded for another 320 s. This unloaded phase is important for the rehydration of the cartilage specimen. Subsequently, the reciprocating test was restarted immediately after reloading and continued for another 300 s. The unloading phase was repeated twice so three tests under the same conditions were performed (Figure 1c). The friction and the normal force were continuously monitored through a biaxial force sensor connected to the pin holder. From these data, the coefficient of friction was calculated. Sliding tests were repeated four times under the same conditions with four different cartilage samples and fresh samples of tested lubricant. Between tests with different lubricants, cartilage samples were immersed in PBS.

Intact cartilage specimens with underlying subchondral bone were prepared from porcine femoral heads (Figure 1b). Porcine femurs were obtained from the local slaughterhouse within a few hours of slaughter. Cylindrical cartilage specimens with diameters of 5.6 mm were extracted from the femoral heads using a hollow drill. Just one cartilage specimen from approximately the same area of the femoral head was extracted from each femur. After preparation, the specimen was stored in the freezer at −20 °C in a PBS solution for no more than 2 weeks. This procedure should slow down the biological degradation of the cartilage tissue. It has also been reported [31] that storing the articular cartilage under these condition does not change its mechanical properties. Half an hour before the experiment, the cartilage was removed from the freezer to thaw at room temperature.

## 3. Results and Discussion

### 3.1. Rheology of HA

**Figure 2:**
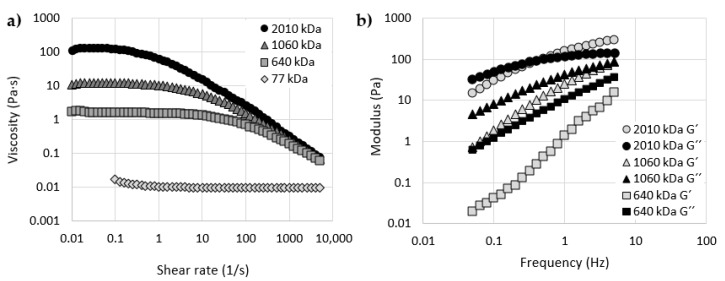
(**a**) Viscosity as a function of shear rate for HA solutions with different molecular weights; (**b**) Elastic (G′) and viscous (G″) moduli as a function of frequency for HA solutions with different molecular weights.

Firstly, the viscosity of all the tested HA solutions was measured. The viscosity curves (a viscosity dependence on shear rate) of all four HA samples with different molecular weights are shown in Figure 2a. The results showed a strong dependency between the viscosity and molecular weight of HA. The highest viscosity was measured for HA with a molecular weight of 2010 kDa and the lowest for 77 kDa HA over the whole range of shear rate. The measured zero shear viscosity (viscosity for the lowest tested shear rate – 0.01 1/s) for all tested samples was (mean value ± standard deviation): 107.1 ± 1.7 Pa·s; 11.6 ± 0.4 Pa·s; 1.67 ± 0.05 Pa·s. The viscosity of 77 kDa HA was not measured at very low shear rates due to the limitations of the experimental methodology. Viscosity was measured on the rheometer in a cone–plate configuration; the measurement of such low viscosity fluid at very low shear rates in this configuration was not possible. The zero shear rate viscosity of this sample was measured at 0.1 1/s and the measured value is 0.013 ± 3 ×10^‒3^ Pa·s. The zero shear rate viscosity of the synovial fluid from a healthy joint ranges from 1 to 175 Pa·s [32], while the zero shear rate viscosity of the synovial fluid obtained from osteoarthritic joint ranges from 0.01 to 11 Pa·s [15,17,32,33]. Based on the zero shear viscosities, tested HA solutions correspond to the osteoarthritic synovial fluid or to the low viscosity synovial fluid from a healthy joint even though the concentration of HA is much higher. Interestingly, studies with commercial viscosupplements also exhibit a large dispersion in results. The zero shear rate viscosity of commercial viscosupplements can vary between 0.5 and 190 Pa·s [34,35]. It can be assumed that these low viscosity HA solutions will not perform well in the recovery of the rheological properties of osteoarthritic synovial fluid after mixing with it. Resumption of the rheological properties of healthy synovial fluid after mixing osteoarthritic synovial fluid with viscosupplement is one of the main objectives of viscosupplementation.

HA exhibited the non-Newtonian shear thinning behavior (viscosity decrease with increasing shear rate). Molecules of HA are entangled and most resistant to flow at low shear rates. At high shear rates, molecules disentangle and align in the shear field. The strongest shear thinning behavior can be observed with 2010 kDa HA. HA with longer polymer chains allow for a greater number of entanglements and consequently for a higher value of zero shear viscosity and a stronger shear thinning behavior [18]. On the other hand, the shear thinning behavior for 77 kDa HA is relatively weak and can be observed in a very small range of shear rates. This HA sample exhibits Newtonian behavior most of the time. For example, the rate of shear thinning behavior can be analyzed by the value of $\frac{\eta_{0}}{\eta_{x}}$, which is the ratio of the zero shear rate viscosity and the viscosity at some defined values of shear rate [18,36]. Calculated values of shear thinning ratio $\frac{\eta_{0}}{\eta_{300}}$ for all four HA samples are stated in Table 1. Shear thinning ratios for synovial fluid from normal joints vary between 70 and 250 or between 5 and 40 for the synovial fluid aspirated from joints with osteoarthritis. For commercial viscosupplements, this ratio ranges between 2.3 and 651.2 [37]. Three out of four tested HA samples are consistent with results of osteoarthritic synovial joint or viscosupplements with low molecular weight HA again.

The second part of the rheological measurements was an analysis of the viscoelastic properties of the tested HA solutions. Figure 2b contains results of the frequency sweep measurements. The graph contains the storage and loss modulus dependency on the frequency of oscillating motion for three HA solutions. Viscoelastic properties of 77 kDa HA were not measurable in the plate–plate configuration. HA with molecular weights of 640 kDa and 1060 kDa exhibited a viscous-like behavior in the whole range of tested frequencies (i.e., the values of G’’ were always higher than the values of G’). Only the results of HA with the highest molecular weight exhibited a viscoelastic behavior, presenting a crossover point at 0.4 Hz. This point indicates a transition from the viscous to elastic behavior. The solution shows the viscous behavior at low frequencies, because the molecular chains can release stress by disentanglement and molecular rearrangement during the period of oscillation. However, at high frequencies, chains cannot disentangle during the short period of oscillating motion; therefore, the solution exhibits elastic behavior [38]. The crossover frequency is also important because it determines to what extent the fluid absorbs or dissipates energy [18]. Balazs [39] reported nearly the same value of crossover point frequency for healthy synovial fluid obtained from the knees of individuals over the age of 52. The crossover point of 0.4 Hz means that, during normal daily activities, such as walking or running (frequency of 0.5 and 2.5–3 Hz [40]), the 2 010 kDa HA solution behaves like the elastic body. It can adsorb mechanical energy and thereby it could protect the articular cartilage against mechanical damage or wear. Values of G’ and G’’ of all tested HA solutions at the frequencies of 0.5 Hz and 2.5 Hz are stated in Table 1. The results showed that the magnitudes of G’ and G’’ increase with the molecular weight of HA and the potential crossover point moves to lower frequencies.

### 3.2. Cartilage Friction Analysis

**Figure 3:**
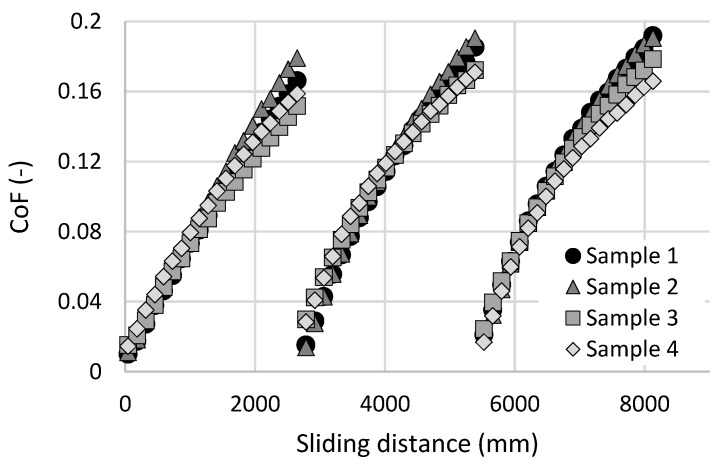
Coefficient of friction as a function of a sliding distance for four cartilage samples lubricated by phosphate buffer saline (PBS).

A distance-dependent frictional behavior for four cartilage samples lubricated by PBS is shown in Figure 3. The initial friction is very low, as is typical for the intact cartilage. The values of the coefficient of friction (CoF) are between 0.01 and 0.015. However, the CoF is gradually increasing with sliding distance. At the end of the first measurement substep (sliding distance of 2740 mm), the values of CoF have increased to 0.15–0.18. This behavior corresponds to the theory of biphasic lubrication by Ateshian et al. [3,24,41]. The friction is strongly influenced by load support from pressurized interstitial fluid (i.e., by exudation and rehydration of the cartilage porous structure during the loaded and unloaded phases of gait cycle). After the sliding distance of 2740 mm, the cartilage specimen was unloaded for 320 s. When the cartilage sample was reloaded and the reciprocating test restarted, friction was significantly decreased from the previous high level. This recovery of low friction was caused by the previously mentioned rehydration of cartilage, but the initial values of CoF are slightly higher than in the first measurement substep. This phenomenon could be caused by insufficient rehydration of the cartilage or by partial removal of the boundary lubricating layer from the cartilage surface. In the second and third measurement substeps, a very similar frictional behavior to the first substep was observed. However, the initial and final values of CoF were slightly higher compared to the previous substep.

**Figure 4:**
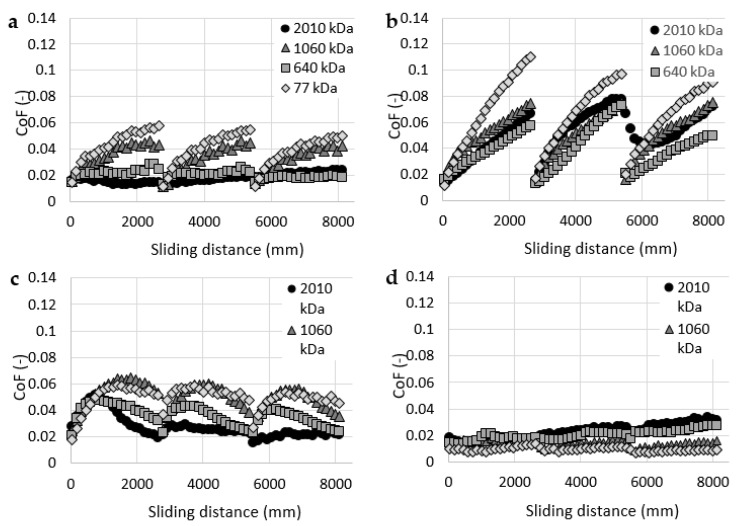
Coefficient of friction as a function of a sliding distance for hyaluronic acid (HA) solutions with a molecular weight of: (**a**) cartilage sample 1, (**b**) cartilage sample 2, (**c**) cartilage sample 3, (**d**) cartilage sample 4.

After this initial set of experiments, all four cartilage samples were tested with all four HA solutions as lubricants. The results of these experiments are stated in Figure 4. Each graph contains data measured with one cartilage specimen and four HA solutions with different molecular weights. The results showed a significant decrease in friction compared to the pure base solution (i.e., PBS). Similar trends were published in studies [4,30,42]. The most significant decrease can be observed with sample 4 (Figure 4d). At the end of the frictional tests, the values of CoF measured with HA with different molecular weights varied between 0.009 and 0.03 compared to the value of 0.16 measured with pure PBS. On the other hand, sample 2 (Figure 4b) exhibited the highest values of CoF. At the end of the frictional tests, values of CoF varied between 0.05 and 0.9 depending on the molecular weight of HA. The highest friction for sample 2 was measured with 77 kDa HA and the lowest friction with 1060 kDa HA. Nevertheless, the friction measured with sample 2 and 1060 kDa HA was still higher than any results of sample 4.

Overall, HA solutions showed a larger scatter of data compared with the rheological measurements, and no clear dependence between the molecular weight (viscosity) of HA and the friction in the cartilage model can be observed. The study by Kwiecinski et al. [43] reported an approximately linear dependency between the HA molecular weight and the coefficient of friction in cartilage-on-cartilage contact. Higher molecular weight of HA leads to lower values of CoF. This trend can be partially observed with sample 1 (Figure 4a) and 3 (Figure 4c). 

**Figure 5:**
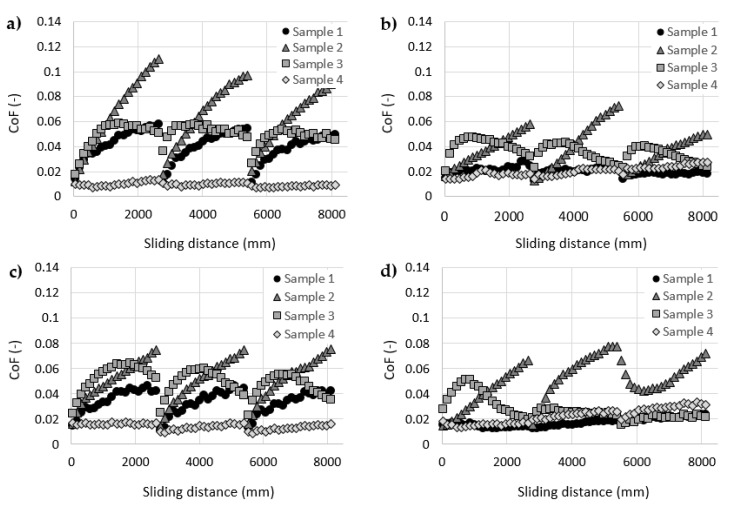
Coefficient of friction as a function of a sliding distance for four cartilage samples and HA solutions with a molecular weight of: (**a**) 77 kDa, (**b**) 640 kDa, (**c**) 1060 kDa, (**d**) 2010 kDa.

Figure 5 contains the same data as Figure 4 but each graph contains data of measurements with all four cartilage samples and one HA solution. The data showed that the HA solution interacts differently with each cartilage sample. Overall, the results showed relatively significant friction differences between cartilage samples. These differences could be affected by differences in the geometry, structure and mechanical properties of cartilage samples. Each cartilage sample was extracted from one porcine femoral head from approximately the same area. However, studies by Appleayrd et al. [44] or by Kiviranta et al. [45] showed a different content of collagen fibrils and proteoglycans across the tibia plateau, patellae, etc. These differences affect the mechanical properties of the cartilage, such as Young’s modulus or Shear modulus. Moreover, Richard et al. [46] reported differences in Young’s modulus and Poisson ratio between the healthy cartilages from six patients with femoral neck fracture only. Samples from four different cartilage areas were tested on each femoral head. These differences in the mechanical properties of the cartilage sample, resp. the location of sample extraction, also affect the friction of the cartilage [47,48]. Some differences in the geometry of samples can also be caused by the methodology of their extraction. 

An important role may also be played by possible interactions between HA and the residues of synovial fluid on the surface of the cartilage. All samples were bathed in PBS prior to the frictional measurements. Solutions, such as sodium dodecyl sulphate or alcohol, are commonly used prior to the biotribological experiments to clean the surfaces of the proteins, phospholipids, etc. [49,50,51]. However, these solutions could possibly damage the structure of the cartilage. Reactions between HA and proteins can be either synergistic or unbeneficial for cartilage friction [29,30,42]. However, the reactions between HA and phospholipids seem to be crucial for the effectiveness of HA in lowering friction [4,27,52]. The theory by Klein et al. [28,53,54,55] assumes that HA may complex with lipids such as Dipalmitoylphosphatidylcholine (DPPC), that are present in the articular cartilage and in the surrounding synovial fluid, to provide a robust boundary layer with extremely low friction.

Different results of every cartilage specimen should also be related to the inconsistent results of viscosupplementation in clinical practice. Some studies report positive effects of viscosupplementation. Maheu et al. [56] report an improvement in the pain or function of osteoarthritic joints up to 40 months after viscosupplementation. Tikiz et al. [57] reported a significant reduction in VAS (Visual Analogue Scale) and WOMAC (Western Ontario and McMaster Universities Osteoarthritis Index) indexes for a period of 6 months in patients with hip osteoarthritis. Nevertheless, authors did not find any significant differences between viscosupplements with high and low molecular weights. On the other hand, many studies did not find differences between HA and anti-inflammatory drugs [58] or placebo [59]. Some of them even report an increased risk of serious adverse event after therapy [60,61]. Ambiguity of results leads to the non-uniform recommendations of international medical associations. For example, the European Society for Clinical and Economic Aspects of Osteoporosis and Osteoarthritis (ESCEO) recommends viscosupplementation for the advanced pharmacological management of knee osteoarthritis in patients who remain symptomatic despite the use of non-steroidal anti-inflammatory drugs [62]. On the other hand, Osteoarthritis Research Society International (OARSI) considers viscosupplementation as uncertain but possible for the treatment of knee osteoarthritis [63].

**Figure 6:**
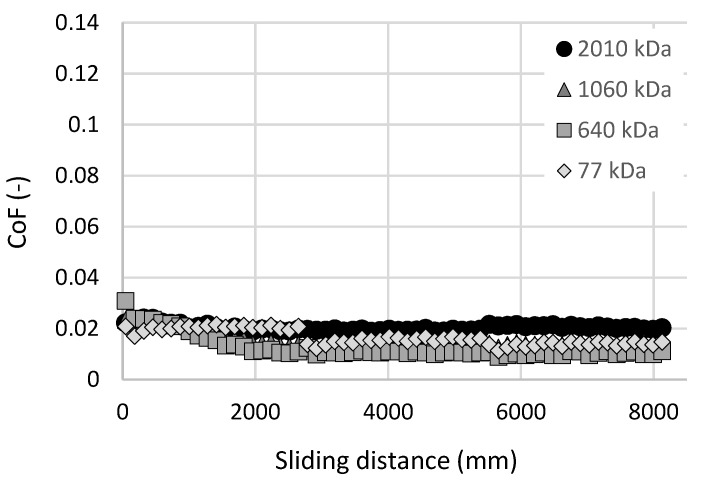
Coefficient of friction as a function of sliding distance for polyvinyl alcohol (PVA) hydrogel-on-glass configuration and HA solutions with different molecular weights.

Experiments with the articular cartilage showed a large dispersion in results. The main reasons are likely to be the differences in structure and shape between the individual cartilage specimens. Therefore, for our future studies on viscosupplementation, a cartilage substitution is one of the possibilities to improve the repeatability of the measurements. The substitutional material should be more homogenous in its mechanical properties but it should still have similar mechanical properties, be porous and exhibit low values of CoF. One option may be the use of hydrogels based on polyvinyl alcohol (PVA). These materials are, among others, developed and tested in the long term as a suitable material for the replacement of damaged osteoarthritic cartilage [64,65,66,67,68]. Figure 6 contains the results of the initial experiments with PVA hydrogel as a cartilage replacement. The test rig, experimental conditions and tested lubricants were the same as before. Samples from freeze-thawing PVA hydrogel were made according to the study by Yarimitsu et al. [29]. The hydrogel sample was replaced after each experiment. Results showed a decrease in CoF during the initial run-in phase. After this, most of the HA solutions exhibited constant friction. Values of CoF were even lower than during the experiments with cartilage samples. Continuously, even in this case, no direct dependence between the HA molecular weight and CoF can be seen.

For a detailed study on the effect of viscosupplementation on the friction of the articular cartilage, more complex lubricants should be tested. Interaction between HA and other constituents plays an important role in the lubrication of the articular cartilage [27,28,29,30]. Therefore, lubricants containing other synovial fluid constituents (proteins, phospholipids, etc.) should be tested. More reliable results may also be obtained from measurements with commercial viscosupplements rather than from experiments with pure HA solutions. The methodology of frictional experiments also has some shortcomings. Constant speed and load during sliding motion do not correspond to the conditions in real human joints. The cartilage-on-cartilage configuration is likely to exhibit lower values of CoF which will be closer to the real joints. However, important findings will allow the in-situ observation of the cartilage-on-glass contact by optical methods, which assumes the transparent material of one of the surfaces. Fluorescent microscopy should be suitable for this application. It allows the study of the behavior of the individual components of complex lubricants and is already used for lubrication analysis of joint replacements in the author’s laboratory [49,69]. The pin-on-plate tribometer for the in-situ observation of cartilage-on-glass contact by fluorescent microscopy is currently under development [70].

## 4. Conclusions

The present paper analyzed the rheological properties of HA solutions with different molecular weights and also the frictional behavior of these solutions in the cartilage-on-glass contact during reciprocating sliding tests. Rotational rheometers were employed in order to analyze the viscosity and viscoelastic properties of HA solutions with molecular weights varying between 77 kDa and 2010 kDa. The pin-on-plate tribometer was later employed to analyze the CoF dependency on time in the cartilage-on-glass contact lubricated by these HA solutions. The main conclusions which emerged from the measured data are summarized in the following points:

- Rheological measurements showed a strong dependency between the molecular weight and the viscosity or viscoelastic properties of HA solutions. HA solutions with higher molecular weights exhibited higher viscosity, dynamic moduli and shear thinning ratio.
- The crossover point was measured only for one of the tested HA samples. Based on the obtained data, it can be assumed that a higher molecular weight of HA leads to lower values of crossover frequency.
- CoF measurements showed a substantial dispersion in the results, showing no clear dependency between the HA molecular weight and the friction in the cartilage-on-glass contact.
- Mechanical properties and overall conditions of individual cartilage samples can significantly affect the effectiveness of HA solutions during the reciprocating sliding motion. In most cases, each cartilage sample exhibited the highest and the lowest values of CoF during measurements with different HA solution.
- Unclear results may support the contradictory conclusions of medical studies whose results are strongly dependent on the individual patient’s conditions. The cartilage condition and composition of synovial fluid can significantly affect the effectiveness of viscosupplementation.
- Different results of rheological and frictional measurements might also show the insufficiency of rheological measurements in the assessment of viscosupplements effectiveness.

## Figures and Tables

**Table 1:** Rheological properties of tested HA solutions.

MW (kDa)	Zero Shear Viscosity (Pa·s)	$\frac{\eta_{0}}{\eta_{300}}$	0.5 Hz		2.5 Hz		Crossover Frequency (Hz)
			G′ (Pa)	G″ (Pa)	G′ (Pa)	G″ (Pa)	
2010	107 ± 1.7	113.9	101 ± 3.5	92.3 ± 4	220 ± 9.5	125 ± 6.3	0.4
1060	11.6 ± 0.4	17.8	13.5 ± 1.5	29 ± 2.5	55.8 ± 5.6	67.5 ± 5.3	-
640	1.67 ± 0.05	4.1	0.4 ± 0.04	5.8 ± 0.03	5.4 ± 0.3	22.2 ± 0.2	-
77	0.013 ± 3 × 10^‒3^	1.3	-	-	-		-
